# Optimum Particle Size of Treated Calcites for CO_2_ Capture in a Power Plant

**DOI:** 10.3390/ma12081284

**Published:** 2019-04-18

**Authors:** Luís Quesada Carballo, María del Rosario Perez Perez, David Cantador Fernández, Alvaro Caballero Amores, José María Fernández Rodríguez

**Affiliations:** 1 Department of Energy and Fuels, Higher Technical School of Mining and Energy Engineers of Madrid, Polytechnic University of Madrid, 14014 Madrid, Spain; luis.quesadac@enel.com; 2 Department of Inorganic Chemistry and Chemical Engineering, Faculty of Sciences, University of Córdoba, 14014 Córdoba, Spain; m.rosario.perez76@gmail.com (M.d.R.P.P.); alvaro.caballero@uco.es (A.C.A); 3 Department of Inorganic Chemistry and Chemical Engineering, School of Engineering Science of Belmez, University of Córdoba, 14014 Córdoba, Spain; david_cantadorf@hotmail.com

**Keywords:** greenhouse gas emissions, carbon dioxide, adsorption, calcite, calcite calcined and rehydrates

## Abstract

This work has analyzed the influence of the particle size of a calcite from a quarry, whether original, calcined, or rehydrated, on the efficiency of CO_2_ capture of the gases emitted in a coal-fired power plant. Three different particle sizes 0.5 mm, 0.1 mm, and 0.045 mm have been studied. The calcination had a minimal effect on the particle size of the smaller samples A1045 and A1M1 (<30 μm). The N_2_ isotherms and the CO_2_ adsorption isotherms at 0 °C showed a very significant increase in the surface of the calcined and rehydrated samples (A15CH, A1045CH, and A1M1CH) with respect to the calcined or original samples. The results obtained showed that the capture of CO_2_ for the sample A1M1, with a smaller average particle size (<30 μm, is the most effective. For the sample A1M1 calcined and completely rehydrated (Ca(OH)_2_), the chemical adsorption of CO_2_ to form CaCO_3_ is practically total, under the experimental conditions used (550 °C and CO_2_ flow of 20 mL min^−1^). The weight increase was 34.11% and the adsorption capacity was 577.00 mg g^−1^. The experiment was repeated 10 times with the same sample A1M1 calcined and rehydrated. No appreciable loss of adsorption capacity was observed.

## 1. Introduction

Up to 55% of greenhouse gas emissions corresponds to CO_2_. Nowadays there is a growing concern in several countries regarding the impact caused by emissions of greenhouse gases such as CO_2_. 

The International Energy Agency (IEA) [1] forecasts that demand for energy will increase 40% by 2030, an average annual increase of 1.5% with over 50% of the increase in demand coming from China and India. The analysis, by the Intergovernmental Panel on Climate Change (IPCC) [2], has indicated that the capture and storage of CO_2_ would contribute between 15 and 55% to the global cumulative mitigation effort until 2100, thus presenting itself as a transition technology that will contribute to mitigating climate change.

Various methods for reducing the amount of CO_2_ in the atmosphere have been discussed, such as reducing the amount of energy used and improved efficiency of energy generating processes, use of non-fossil fuels (renewable energy), CO_2_ capture, and sequestration.

The capture of CO_2_ is part of the set of technologies that try to separate CO_2_ from large emission sources (thermal power plants, cement plants, steel mills, ceramics, etc.) and prevent their emission into the atmosphere. The first solutions for the capture of CO_2_ implied the need to inseparably link the catch with transport and geological storage (capture, transport and geological storage technologies (CCS) [3]), the latest research on these technologies is directed towards the generation of a transportation network and geological storage of CO_2_. This network would unite different geographical points of geological storage with enough capacity and would allow decoupling the capture of CO_2_ from transport and geological storage.

Bui et al. (2018) [4] have reviewed the current status of CO_2_ capture, transport, utilization, and storage from a multi-scale perspective. Deployment of large-scale CCS projects has been slow. Reiner 2016 [5] indicated that the implementation of CCS technologies worldwide will require a portfolio of large-scale demonstration projects. Of the 37 major large-scale CCS projects, 17 of these are in operation, four in construction and the remainder are in varying stages of development [4,6]. In addition, Bui et al. (2018) [4] have contextualized CCS technologies in integrated evaluation models (IAM). In order to limit global warming, negative emission (NET) technologies consisting of combining CCS with low-carbon or carbon-free Bioenergy (BECCS) [4,7] are considered key. BECCS has the double benefit of mitigating emissions and generating energy, which makes it attractive from the perspective of cost optimization of an IAM.

Moreover, the knowledge of thermodynamic and transport properties of CO_2_-mixtures is important for designing and operating different processes in carbon capture and storage systems (Tan et al. (2016) [8]).

Other capture technologies consist of giving use to captured CO_2_, these are the so-called carbon capture and utilization (CCU) technologies [9]. One of the advantages of CCU over CCS is that utilization of CO_2_ is normally a profitable activity as products can be sold. In addition, storage potential may be limited or away from CO_2_ sources and public resistance to geological storage of CO_2_ has been noted [10]. Baena-Moreno et al. (2015) [11] presented a list of technologies for Carbon Capture and Utilization (CCU) from laboratory scale to commercially established CO_2_ applications, such as raw materials for the manufacture of fuels, polymers, Chemicals, or as recovery agents in techniques such as Enhanced Oil Recovery (EOR) and Enhanced Coal Bed Methane (ECBM). These technologies not only seek to reduce the volume of CO_2_ emissions into the atmosphere, but also obtain a benefit using CO_2_ in different types of industrial processes replacing conventional raw materials and reducing the consumption of fossil fuels.

Another form of CCU would be biological carbon mitigation (BCM), which consists of the capture of CO_2_ by photosynthetic organisms to convert it into organic carbon using solar energy and producing a significant amount of biomass. BCM is a way to sequester CO_2_ while producing biofuels and valuable by-products. Microalgae serve as a promising agent for carbon sequestration [12,13].

Among the capture systems proposed by the report of the Intergovernmental Panel on Climate Change (IPCC) published in 2005 [3], the most mature and widely used post-combustion capture technology is chemical absorption with monoethanolamine (MEA), which however is an important contributor to CO_2_ emissions and global warming. Therefore, one of the proposed solutions would be the search for more environmentally sustainable adsorbents (Cuellar et al. (2015) [9]). Bui et al. (2018) [4] highlight three aspects of the post-combustion capture of the calcination/carbonation cycles with calcite compared to other technologies: one, that the carbonator / calciner can serve as a heat source for a steam cycle to produce energy additional, another, that the absorber used, calcite, is available in industrial quantities and is a non-hazardous chemical, and finally, that there is the possibility of using spent absorbent in industrial processes such as cement manufacturing, with the consequent decarbonisation partial of this industry or can even achieve near zero emissions by incorporating this technology into its manufacturing process. Calcium looping (CaL) technology has also been progressed to pilot scale. There are two major demonstration projects, one at the University of Darmstadt, Germany [14], and one in La Pereda, Spain [15].

CO_2_ is normally accompanied, in the gas stream produced from the combustion of coal, by SO_2_, NO_x_, and O_2_. The use of the carbonation reaction of CaO to CaCO_3_ as a possible separation reversible process of CO_2_, is adequate, although CaO will also react with SO_2_ to form CaSO_4_ [3]. Calcination, to regenerate the adsorbent, takes place at high temperatures, at around 900–950 °C. In carbonation, a flow of CO_2_ gas is in contact with CaO, at temperatures above 600 °C, with the carbonation reaction being carried out to form CaCO_3_. [16]. The maximum carbonation capacity of CaO decays with the number of carbonation/calcination cycles down to 20% [17,18]; it is necessary to indicate that these authors used particle sizes greater than 100 μm.

Reactions of CaO with SO_2_ could be a problem in the calcination/carbonation cycles since CaSO_4_ does not decompose at the calcination temperature [19]. This implies an accumulation of CaSO_4_ in the cycles, which reduces the efficiency of carbonation [20]. The separation of CaSO_4_ is only possible through a purge with a consequent loss of sorbent [19]. Consequently, SO_2_ should be removed from the gas stream prior to the incorporation of the gas stream into the calcination/carbonation cycles. This is now usual in coal-fired power plants, since by regulation (DEI, 2010) [21], they have to incorporate a desulphurization system. Consequently, if the SO_2_ concentration is very low, the residence time is short, and the temperature remains below 640 °C [22], the carbonation reaction will take place with the sulphation reaction being unlikely.

Different studies about reversibility show the carbonation is far from being reversible [11,12]. After a very fast, and chemically controlled initial reaction, there is a much slower second phase controlled by diffusion into CaCO_3_ layers [23,24,25]. Several papers [20,23,26,27,28] show a decrease in the carbonation maximum of lime with the number of cycles and this has been related to sintering within the material. 

Several authors [29,30,31,32] have shown that the formation of a dense, non-porous layer of carbonate minerals takes place around the reagent particles, so that total carbonation efficiency cannot be attained, except for cases of high relative humidity and small particle sizes (nanometric scale). This protective layer causes a decrease in porosity when the materials are partially carbonated [31,32,33]. This is in agreement with the poor results obtained by Escosa et al. [19] regarding the reaction between CaO and CO_2_ that requires the introduction of much more CaO than is necessary stoichiometrically.

Carbonation can be improved under the action of water at moderate temperature and low CO_2_ pressure [34]. The hydration of CaO with water vapor is typically used to reactivate its performance as a sorbent [35]. In this sense, Maretic, et al. [36], indicated that carbonation was much greater when Ca(OH)_2_ is directly carbonated at high temperatures compared to carbonation of CaO. The carbonation reaction of nanoparticles [35] that takes place is:

Ca(OH)_2(s)_ + CO_2(g)_ = CaCO_3(s)_ + H_2_O_(g)_ (1)

The molecular water produced in the reaction is not stable at the hydroxide-carbonate interface [35]. This assumption is only valid for small Ca(OH)_2_ particles (<30 μm) [37]. Consequently, the results show that the simultaneous expulsion of the molecular water, produced during the carbonation of Ca(OH)_2_, could significantly improve the transfer of CO_2_ from the gas phase to the surface of the unreacted Ca(OH)_2_. If CaO is reacted with CO_2_ at the very moment of the decomposition of Ca(OH)_2_, with particle size less than 30 μm, the adsorption is at a maximum [35].

In order to develop good CO_2_ adsorbents at high temperature several aspects need to be investigated: understanding the mechanism of CO_2_ adsorption on different kinds of adsorbents, finding the best adsorbent for modifying chemical reagent to improve the adsorption capacity; study of the adsorption and desorption kinetics of CO_2_ in the adsorbent and the choice of optimal conditions for the industry [38]. 

The present work deals exclusively with the capture phase, leaving open any of the following possibilities of action with the captured CO_2_, from its transportation and geological storage, to which it no longer has to be linked in an inseparable way, since it can be part of a transport network towards different possibilities of confinement or it can be used with good performance in any of the possible alternatives described by Baena-Moreno et al. (2015) [11].

This paper is intended to address CO_2_ capture using natural limestone (CaCO_3_) of different granulometries, calcined limestone (lime (CaO)) and calcined and rehydrated limestone (portlandite (Ca(OH)_2_)). Among other aspects, the particle size variation in several adsorption-desorption cycles and how this affects the adsorption-desorption temperature and the amount of CO_2_ adsorbed is discussed. Finally, the stability of the adsorbent samples after a large number of adsorption-calcination cycles is considered.

## 2. Materials and Methods 

Nine samples were analysed. The first three A15, A1045, A1M1, are from 0–6000 µm size fraction of crushed limestone from the quarry “Horcallana” owned by Endesa (Teruel, Spain) (sample A1). The sample A15 corresponds to less than 500 µm size fraction of sample A1. A1045 sample corresponds to the particle size less than 45 µm from the sample A1. The A1M1 sample corresponds to the size fraction below the 100 µm A1 sample. The samples were subjected to grinding in a laboratory mill with porcelain balls (45 balls of 30 mm in diameter and each weighing 28 g) for 4 h. 

**Scheme 1:**
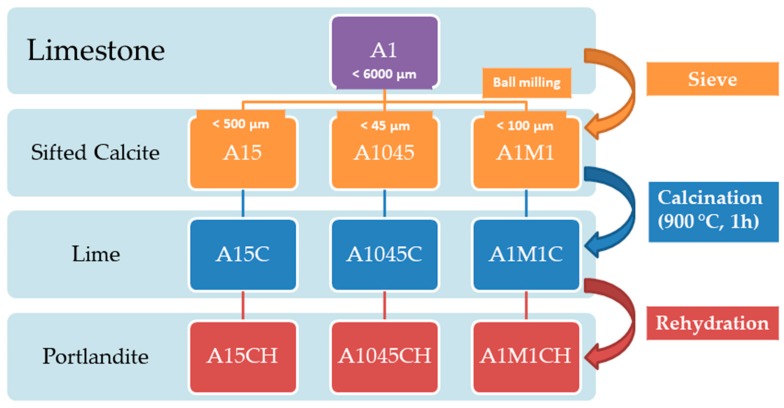
Nomenclature and processes of the samples.

Three uncalcined samples were calcined at 900 °C for 1 h. Samples were identified by adding a “C” to the name of the uncalcined sample (A15C, A1045C, A1M1C). Then, three samples were partially rehydrated in an atmosphere rich in water. Samples were identified by adding an "H" to the name of the calcined sample (A15CH, A1045CH, and A1M1CH). In Scheme 1, the nomenclature and processes of the samples are summarized.

PXRD patters were recorded in a Siemens D-5000 instrument (Billerica, MA, USA), using CuKα radiation (λ = 1.5418 Å) working at a voltage of 40 kV and a current 30 mA. The detector is a scintillation counter with a slit of 1 mm, employing a scan rate of 2° min^−1^ using a step of 0.2° every 0.6 s and sweeping angles between 10 and 80° in units of 2θ.

Particle size data were obtained in a particle size analyser by laser diffraction technology 2000F Mastersizer (Malvern, UK) with one dry dispersion unit Venturi Scirocco 2000 of Malvern Instruments (Malvern, UK). 

The thermogravimetric analysis was carried out in a Setaram Setsys Evolution 16/18 apparatus (Caluire, France), at a heating rate of 5 °C min^−1^ in air.

N_2_ isotherms were determined on an Autosorb iQ (Quantachrome) (Boynton Beach, FL, USA), with samples being degassed at 150 °C under vacuum for 2 h prior to this. The surface was calculated by applying the BET (Brunauer, Emmett and Teller) method in the range of relative equilibrium pressure 0.05 ≤ P/P_0_ ≤ 0.20 [39]. The porosity distribution was determined by a density functional theory (DFT) method using a cylindrical model for N_2_ on an oxide surface. 

Microstructural characterisation of the materials was carried out using JEOL 1400 TEM (Jeol, Tokyo, Japan).

The CO_2_ adsorption studies were carried out in a Setaram Setsys Evolution 16/18 apparatus (Caluire, France). 40.00 mg of sample was introduced into the equipment and it was evacuated before a CO_2_ flow of 20 mL min^−1^ was passed for one hour, with the thermogravimetric analysis being carried out up to a temperature of 1000 °C.

Finally, CO_2_ adsorption isotherms were recorded on a Micromeritics ASAP 2020 apparatus (Norcross, GA, USA). Commonly used to obtain the BET isotherms of N_2_, which has the possibility of using CO_2_ adsorbate gas in its software, using samples previously outgassed at 150 °C under vacuum for 2 h. The CO_2_ adsorption isotherms were carried out at 0 °C between the values of 0–0.035 of P/P_0_. 

**Scheme 2:**
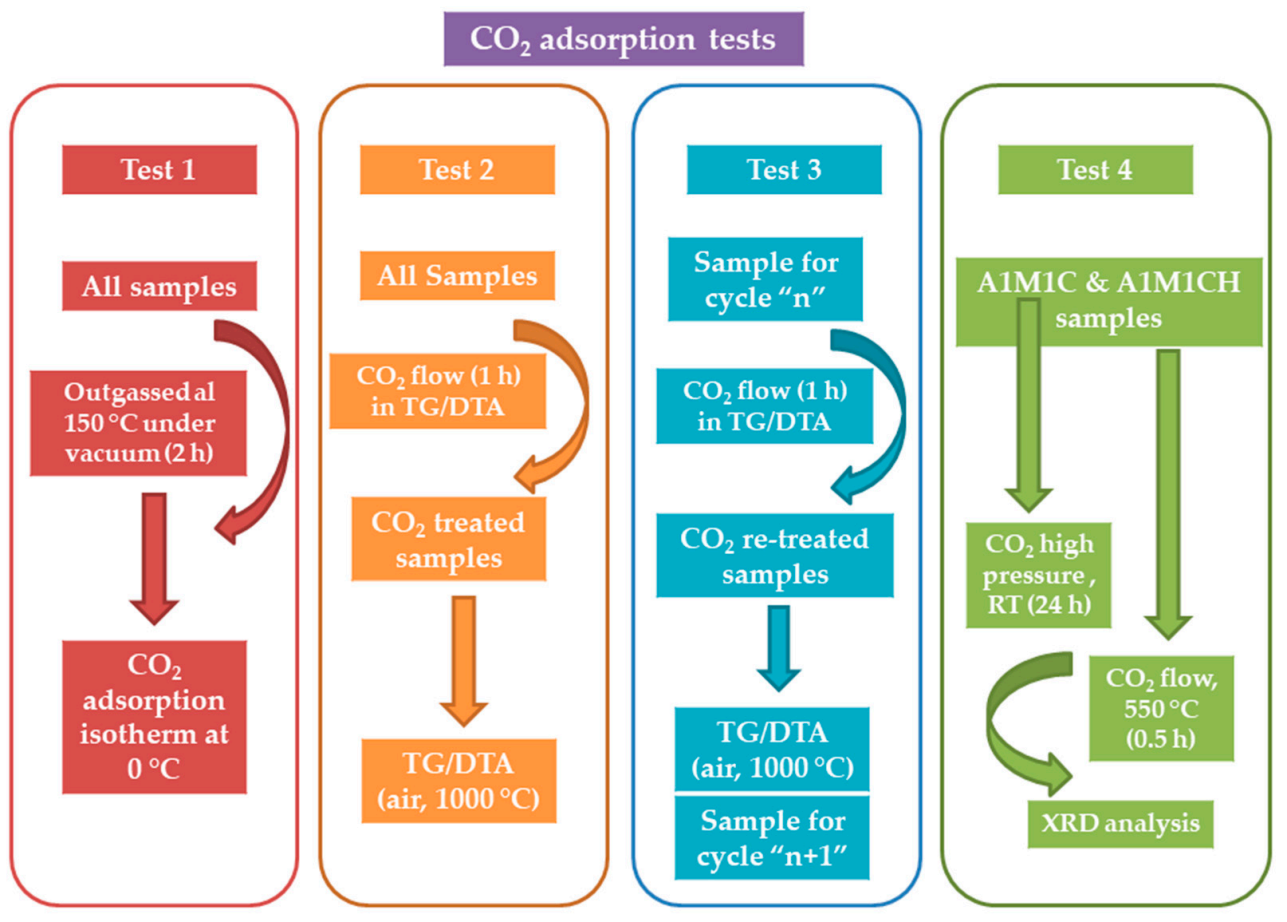
Procedures for the study of CO_2_ adsorption in different samples.

The study of CO_2_ adsorption in the different samples was carried out by means of four different procedures (Scheme 2). In the first, the CO_2_ adsorption isotherms were performed in a Micromeritics ASAP 2020 apparatus, commonly used to obtain the BET isotherms of N_2_, which has the possibility of using CO_2_ adsorbate gas in its software. In the second, the samples were subjected to a CO_2_ flow of 20 mL min^−1^ for 1 h in a thermogravimetric analysis equipment (ATD-TG) (Caluire, France) and later a thermogravimetric analysis (ATD-TG) was carried out in an air atmosphere up to 1000 °C. In the third, successive adsorptions of CO_2_ were carried out to determine the fall of the adsorption capacity after a certain number of cycles. For this procedure the sample, calcined in a previous step, was placed inside in the TG/DTA apparatus and then the sample was treated with a CO_2_ flow of 20 mL min^−1^ for one hour and finally, thermal analysis up to 1000 °C was conducted. The fourth procedure consisted of the simultaneous heating of two samples, one calcined and one totally hydrated, in a tubular oven in a CO_2_ atmosphere produced by a CO_2_ flow of 20 mL min^−1^ during the whole calcination process (1/2 h) at a temperature of 550 °C.

## 3. Results and Discussion

### 3.1. Characterization of Samples

**Figure 1:**
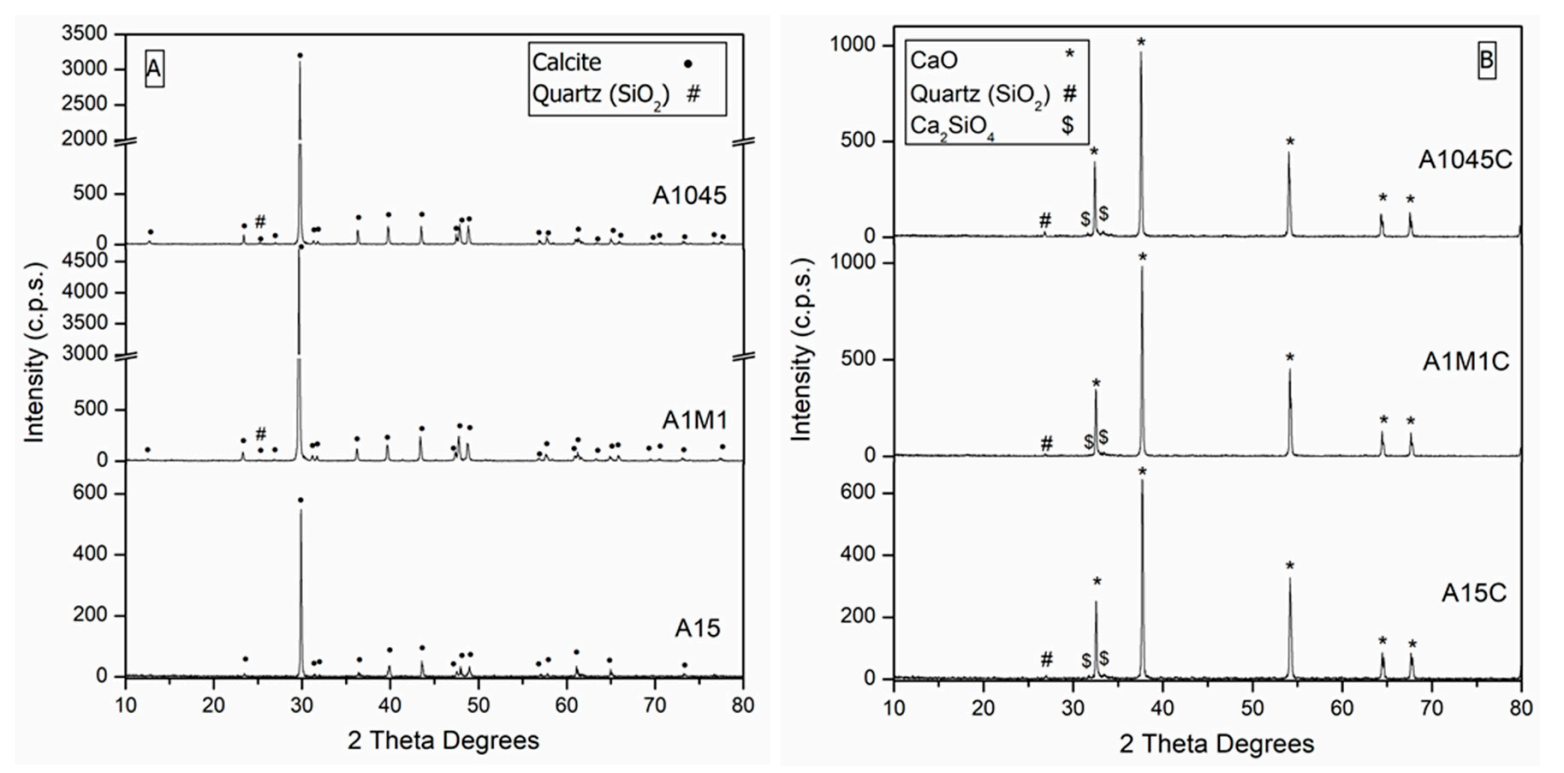
X-ray diffraction (XRD) patterns. (**A**) calcites: A1045, A1M1, and A15; (**B**) calcites calcined: A1045C, A1M1C, and A15C.

The X-ray diffraction diagrams of the original samples without calcining (Figure 1A), shows the presence of calcite (JCPDS: 05-0586) [40] as a main phase, with a negligible amount of impurities of SiO_2_ (JCPDS: 46−1045) [40]. In the samples calcined at 900 °C for 1 h (Figure 1B), CaO was the main phase detected (JCPDS: 37−1497) [40] with negligible amounts of SiO_2_ (JCPDS: 46−1045) [40] and Ca_2_SiO_4_ (JCPDS: 33-0303) [40].

**Figure 2:**
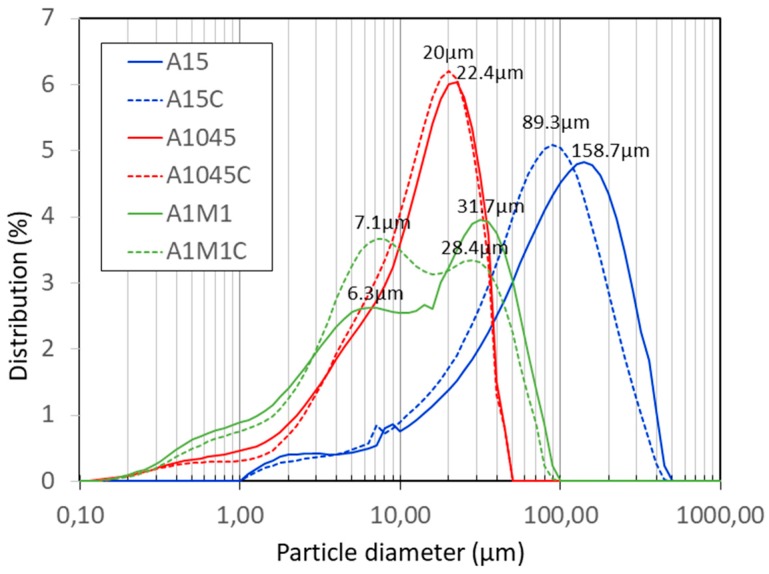
Grain-size distribution of calcites: A1045, A1M1, A15 and calcites calcined: A1045C, A1M1C, and A15C.

Figure 2 shows the particle size distribution curves of the original and calcined samples. In the sample A15, it can be seen that the calcination leads to a considerable decrease in the particle size, with the maximum of the particle size distribution decreasing from around 158 to around 89 μm. The calcination had a minimal effect on the sample size A1045 (maximum at around 22 μm). The sample A1M1, subjected to double-milling, has a bimodal distribution of the particle size, with its average size being the lowest of all (19 μm). Calcining gives rise to an increase in the proportion of smaller particle size, although the average distribution shows little change, around 18 μm. In this case, as well, the distribution peak of larger particle size is around 28 μm.

**Figure 3:**
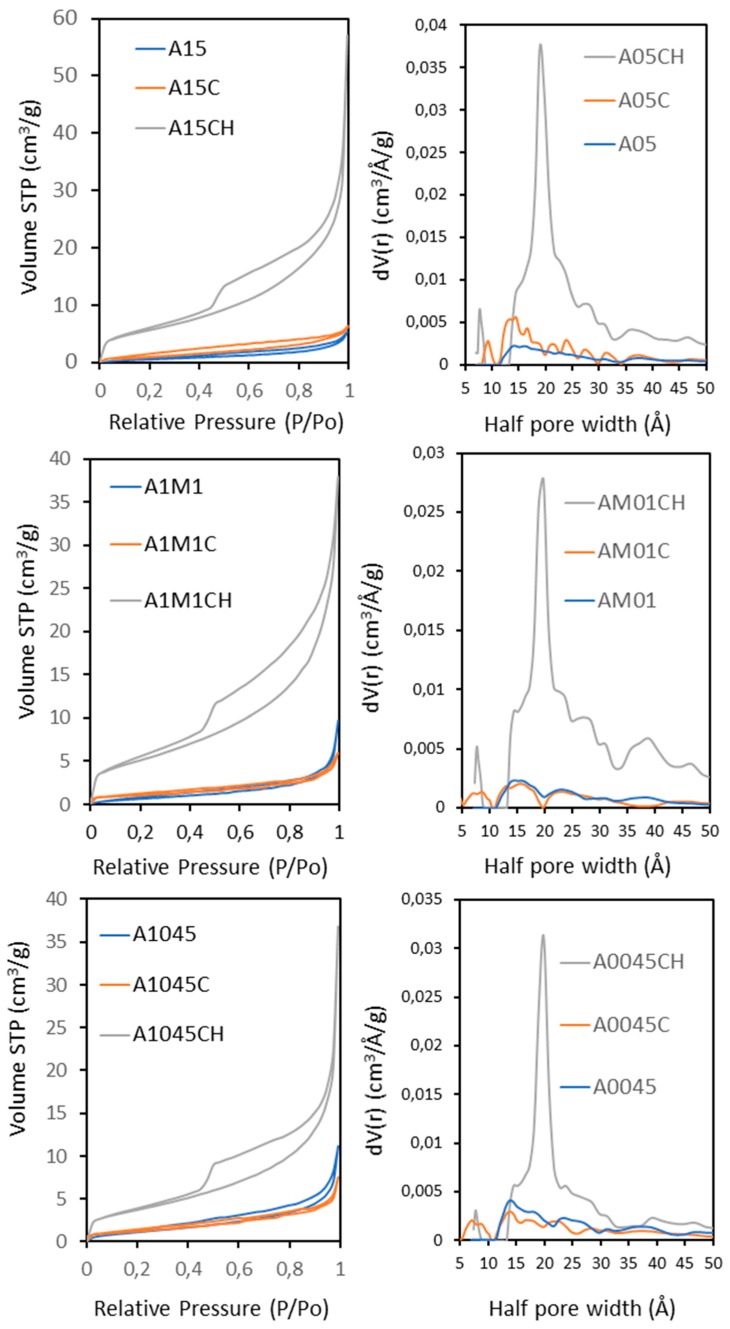
(Left) Nitrogen adsorption–desorption isotherms for calcites: A1045, A1M1, and A15; calcites calcined: A1045C, A1M1C, A15C and calcites calcined and rehydrated: A1045CH, A1M1CH, and A15CH. (Right) Pore size distributions for calcites: A1045, A1M1, and A15; calcites calcined: A1045C, A1M1C, A15C, and calcites calcined and rehydrated: A1045CH, A1M1CH, and A15CH.

Figure 3 (left) shows the N_2_ adsorption isotherms of the three original samples (CaCO_3_), calcined (CaO) and rehydrated to Ca(OH)_2_. Their isotherms were of type IV with a sharp increase in adsorption at P/P_0_ between 0.05 and 0.45 due to capillary condensation, typical of materials with a narrow distribution in the mesopore range (Figure 3 (right)). For the three calcined and rehydrated samples, a sharp capillary condensation step at P/P_0_ = 0.45 was observed. All samples present a hysteresis loop of type H3 or H4, which does not exhibit any limiting adsorption at high P/P_0_, related to aggregates of plate-like particles. The three calcined and rehydrated samples present a hysteresis loop of type H3 with slit-shaped pores. The rest of the samples present a hysteresis loop of type H4 often associated with narrow slit-like pores. This is in accordance with the pore size distributions (Figure 3 (right)). The main physical properties of the three adsorbents are summarized in Table 1. The specific surfaces obtained were 2.7, 4.7, and 3.1 m^2^ g^−1^ for the samples A15, A1045 and A1M1 respectively, and 4.4, 4.5 and 3.9 m^2^ g^−1^ for the calcined samples A15C, A1045C, and A1M1C, respectively. While the surface area of the three calcined samples is very similar, the largest initial average size increase was for the A15 sample, the one that has the largest initial size. A very significant increase is observed in the calcined and rehydrated samples A15CH, A1045CH and A1M1CH, of 21.3, 13.8 and 18.9 m^2^ g^−1^, respectively. This results in a significant increase in pore volume of 8.84×10^−2^, 5.70×10^−2^ and 5.87×10^−2^ cm^3^ g^−1^, respectively (Table 1 and Figure 3 (left)). The average pore diameter was calculated between 2.77–3.97 nm (Table 1 and Figure 3 (right)). It is important to note that in the two calcinated and hydrated samples of smaller size, the same average pore diameter value (3.97 nm) is observed. A negligible micropore content can also be observed for the calcined samples and for the calcined and rehydrated ones (Figure 3 (right)).

### 3.2. CO_2_ Adsorption Studies

**Figure 4:**
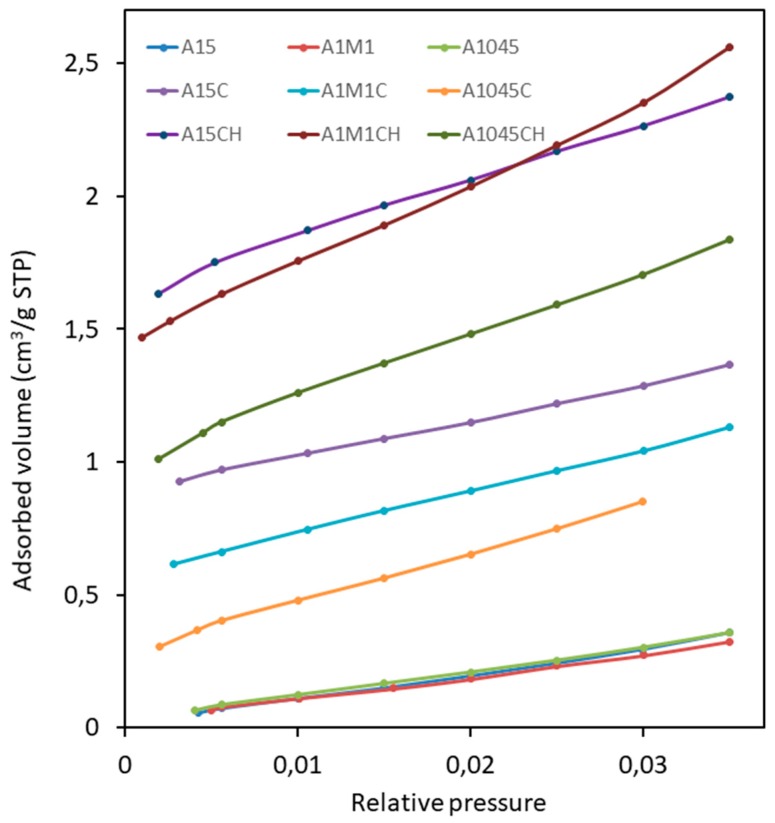
CO_2_ adsorption–desorption isotherms for calcites: A1045, A1M1, and A15; calcites calcined: A1045C, A1M1C, A15C, and calcites calcined and rehydrated: A1045CH, A1M1CH, and A15CH.

In order to delve further into the possibilities of adsorption of original, calcined or hydrated calcites, in line with its possible use for capturing CO_2_ in a power plant, four tests were carried out that allow us to elucidate which is the optimum particle size, as well as the conditions of adsorption. In the first test, the CO_2_ adsorption isotherms were conducted at 0 °C between the values of 0–0.035 of P/P_0_. In all the calcined samples (A15C, A1045C, and A1M1C) an increase of the equivalent surface area is observed, with respect to the samples without calcining (A15, A1045, and A1M1), and especially in the partially calcined and rehydrated samples (A15CH, A1045CH and A1M1CH) (Table 1). These results show a greater capacity of CO_2_ adsorption of the A1M1CH sample (5.3 mg of CO_2_ g^−1^ of adsorbent) (Figure 4), which is in accordance with the highest value of equivalent surface (19.1 m^2^ g^−1^) (Table 1). The results obtained show that the capture of CO_2_ for the sample with a smaller average particle size, A1M1, is more effective. To complete these results, an additional experiment was carried out at room temperature and high CO_2_ pressure, which introduced 50.00 gr of freshly calcined A1M1 sample (at 900 °C for 2 h in an air atmosphere) in a three-litre volume, metal bottle, for 24 h at room temperature and at a pressure of 23 bars of CO_2_. Subsequently, the solid was extracted and an increase of 0.375 g (0.75%) was noted. In short, 7.9 mg of CO_2_ g^−1^ of adsorbent was adsorbed, which is consistent with the results obtained by other authors, e.g., Yong et al. [38]. XRD diagrams (not shown) confirmed that the only phase present before and after the experience was CaO, which it is in accordance with the physical nature of the adsorption.

In tests 2 and 3 we analyzed the effect of high temperature and the effect of adsorption-calcination cycles on the capacity of CO_2_ adsorption. 

**Figure 5:**
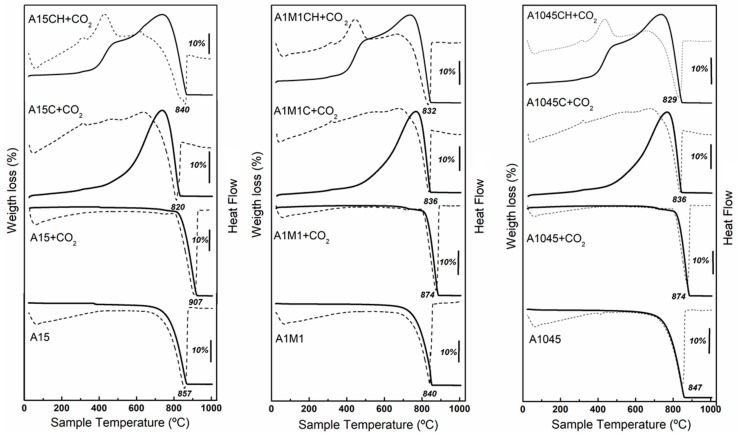
TG (solid lines) and DTA (dotted line) curves: for calcite A15, calcite A15 + CO_2_, calcite calcined A15C + CO_2_ and calcite calcined and rehydrated A15CH + CO_2_; for calcite A1045, calcite A1045 + CO_2_, calcite calcined A1045C + CO_2_ and calcite calcined and rehydrated A1045CH + CO_2_; for calcite A1M1, calcite A1M1 + CO_2_, calcite calcined A1M1C + CO_2_ and calcite calcined and rehydrated A1M1CH + CO_2_.

The results of the second test are shown in Figure 5 and in Table 2. In calcite samples that were not subjected to a flow of CO_2_, an endothermic effect is observed, which is associated with the transformation of calcite (CaCO_3_) in lime (CaO), centred around 840–857 °C. A slight decrease in the decomposition temperature is observed as the average size of the particles decreases (Figure 2). In all the samples subjected to a current of CO_2_, a stabilization of the calcite is observed since it slightly increases the temperature of the endothermic effect, 874–907 °C., being larger in samples of larger particle size. This is related to the equilibrium between the CO_2_ in the atmosphere surrounding the particulates and the mineralized CO_2_ on the surface thereof. The reaction will only proceed if the partial pressure of CO_2_, in the gas above the solid surface, is less than the decomposition pressure of the CaCO_3_ [41,42].

For the calcined samples (A15C, A1045C, and A1M1C) exposed to a current of CO_2_, a continuous weight gain in a single stage is observed which correlates with the formation of CaCO_3_. In the calcined and partially rehydrated samples (A15CH, A1045CH, and A1M1CH) a weight increases in two stages, related to the formation of CaCO_3_ is seen. The final mass is lower than the starting mass and the difference in weight correlates with the water loss from Ca(OH)_2_ in its transformation to CaO.

The results obtained for the calcined samples (A15C, A1045C and A1M1C) are in agreement with those obtained for non-isothermal conditions by Montes-Hernandez et al. [35], although lower than those obtained for these authors. This is because the experimental work conditions used were different, Montes-Hernandez, et al. [35] maintained a CO_2_ flow of 50 mL min^−1^ throughout the experiment. Total carbonation is achieved at temperatures above 850 °C. In the present case, the greatest increase in weight, due to Equation (2), was somewhat less than half the theoretical maximum amount according to Equation (2). In other words, 42.11% of the total CaO was carbonated. The total adsorption of CO_2_ was greater in the A1M1C sample and corresponds to a value of 330.5 mg of CO_2_ g^−1^ of adsorbent.

CaO_(s)_ +CO_2(g)_ → CaCO_3(s)_ (2)

Regarding the calcined and partially rehydrated samples (A15CH, A1045CH, and A1M1CH), the difference between the total weight increase and the total weight loss enables the calculation of the amount of water corresponding to Ca(OH)_2_ and therefore, the amount of Ca(OH)_2_ and CaO present in the samples. The increase in weight in the first stage corresponds to Equation (1), with the largest increase occurring in the sample of smaller average particle size A1M1CH, 17.57%. In the A15CH and A1045CH samples, the increase was 14.56 and 14.59, respectively.

This reaction assumes the simultaneous release of molecular water from Ca(OH)_2_, and the incorporation of CO_2_ into the particles with the subsequent carbonation, mainly for small particles of Ca(OH)_2_ (<30 μm), Blamey et al. [37]. In the present work, this process occurs even for sizes up to 89 μm (sample A15C), although the adsorption of CO_2_ is somewhat lower. This behavior seems to be in accordance with that established by Marsh and Ulrichson [22] that indicates that the hydration of CaO to Ca(OH)_2_ and subsequent calcination to CaO, gives rise to a much more porous adsorbent.

The second weight increase corresponds to the carbonation reaction of CaO (Equation (2)). The total weight increase was greater in the A1M1CH sample (28.39%). Subsequently, the decomposition of the formed calcite (CaCO_3_), occurs at a temperature slightly lower than that of the original samples. These results are in agreement with the studies carried out by Montes et al. [35] that showed that the carbonation of CaO occurs in a wide range of temperatures (100–900 °C) and that of Ca(OH)_2_ occurs in a single narrow range of temperatures (500–600 °C). The adsorption was lower than those obtained by Montes-Hernandez et al. [35]. This is because the experimental work conditions used by these authors were different, maintaining a CO_2_ flow of 50 mL min^−1^ throughout the experiment. The total adsorption of CO_2_ was greater in the A1M1CH sample and corresponds to a value of 368.44 mg of CO_2_ g^−1^ of adsorbent showing that approximately, 32% of the total CaO has reacted. The presence of CaO partially hydrated (A1M1CH) favors the adsorption of CO_2_, compared to the unhydrated sample (A1M1C) for which a value of 330.5 mg of CO_2_ g^−1^ of adsorbent was obtained. Further, that is in accordance with the surface increase (S_BET_) and of pore volume (V_p_) (Figure 1) that occurs when hydrating of the sample A1M1C. That concurs with the results commented by Marsh and Ulrichson [22].

The mechanism of carbonation has been proposed by different authors (Barker [23], Bhatia [24], Mess [25], and Abanades [16]). After a very rapid, chemically controlled, initial reaction period a much slower second stage is followed, controlled by diffusion in the CaCO_3_ layers. It is for this reason that the smaller the particles, the faster the diffusion stage and the deeper the carbonation, reaching, in some cases, the total carbonation of the particles.

**Figure 6:**
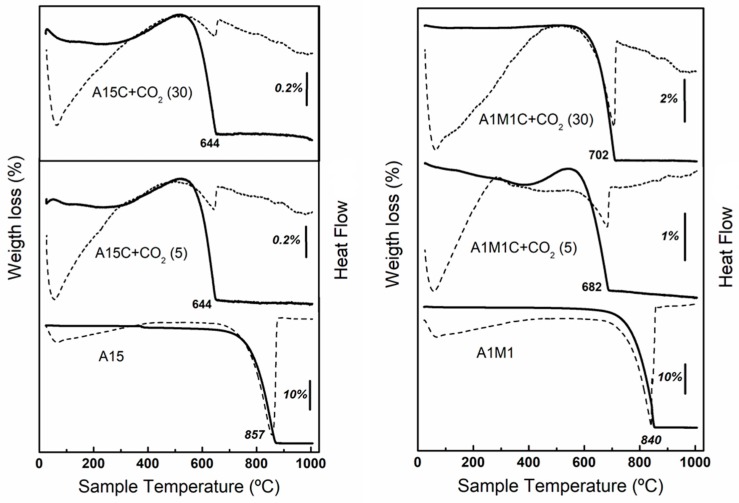
TG (solid lines) and DTA (dotted line): (left) curves for calcite A15, calcite A15 after 5 cycles and calcite A15 after 30 cycles of calcination—CO_2_ flow of 20 mL min^−1^ (1 h)—calcination in air a 900 °C; (right) curves for calcite A1M1, calcite A1M1 after 5 cycles and calcite A1M1 after 30 cycles of calcination—CO_2_ flow of 20 mL min^−1^ (1 h)—calcination in air a 900 °C.

**Figure 7:**
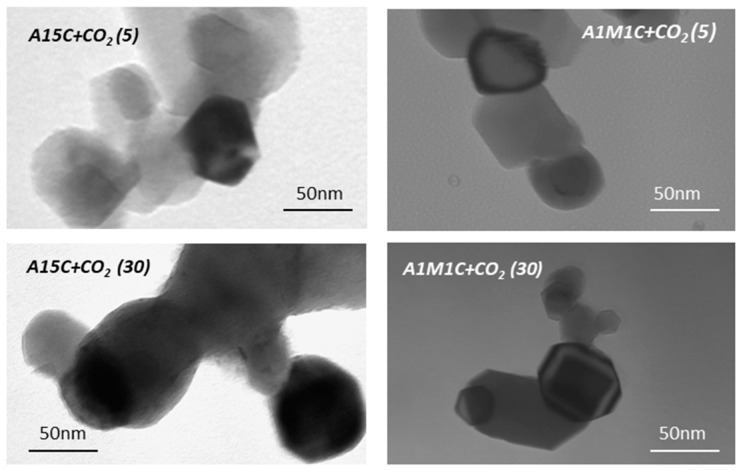
TEM images for calcites A15 and A1M1 after 5 and 30 cycles of calcination—CO_2_ flow of 20 mL min^−1^ (1 h)—calcination in air a 900 °C.

The third test to be analyzed was the loss of adsorption capacity after several cycles adsorption-calcination in an ATD-ATG apparatus. The samples with larger and smaller particle sizes, A15 and A1M1, were chosen for analysis to see if particle size would play an important role in the adsorption process. Figure 6 include the ATD-TG diagrams of the A15 and A1M1 samples after 5 and 30 cycles. After the calcination process of each cycle, the only phase present was CaO (X-ray diffraction diagrams not shown). According to the ATG curves, there is a considerable reduction in the temperature at which the elimination of CO_2_ takes place, from 857 °C to 644 °C (Figure 6 left) and from 840 °C to 682 °C (Figure 6 right), compared to the original samples after 5 cycles, and to 644 °C (Figure 6 left) and 702 °C (Figure 6 right) after 30 cycles. Likewise, the results show that repeated calcination at 900 °C, without previous hydration steps, reduced the capacity of CO_2_ adsorption considerably. This effect is less pronounced (Figure 6 right) when the particle size is smaller (Figure 2). In this case, the A1M1C sample, after 30 cycles, retains an adsorption capacity of 3.83% (30 mg of CO_2_ g^−1^ of adsorbent), higher than that of sample A15C, which is only 0.55% (4.3 mg of CO_2_ g^−1^ of adsorbent). This agrees with the transmission microphotographs (Figure 7) of A15C and A1M1C samples after 5 and 30 cycles. In all cases, homogeneous layered particles with hexagonal habits were observed, with low porosity and slightly larger grain size for A15C sample. These results are in agreement with the studies carried out by Abanades [16], Abanades and Alvarez [17], Grasa, et al. [18], and Blamey et al. [43] who observed that the initial capacity of limestone adsorption/desorption after multiple cycles fell to an asymptotic value as a consequence of sintering processes. Furthermore, the lower the temperature is, and the less time spent in the calciner, the less the sintering occurs. These results seem to indicate that, after the first adsorption-calcination cycle, the calcination temperature decreased to around 700 °C, which reduces the risk of sintering.

**Figure 8:**
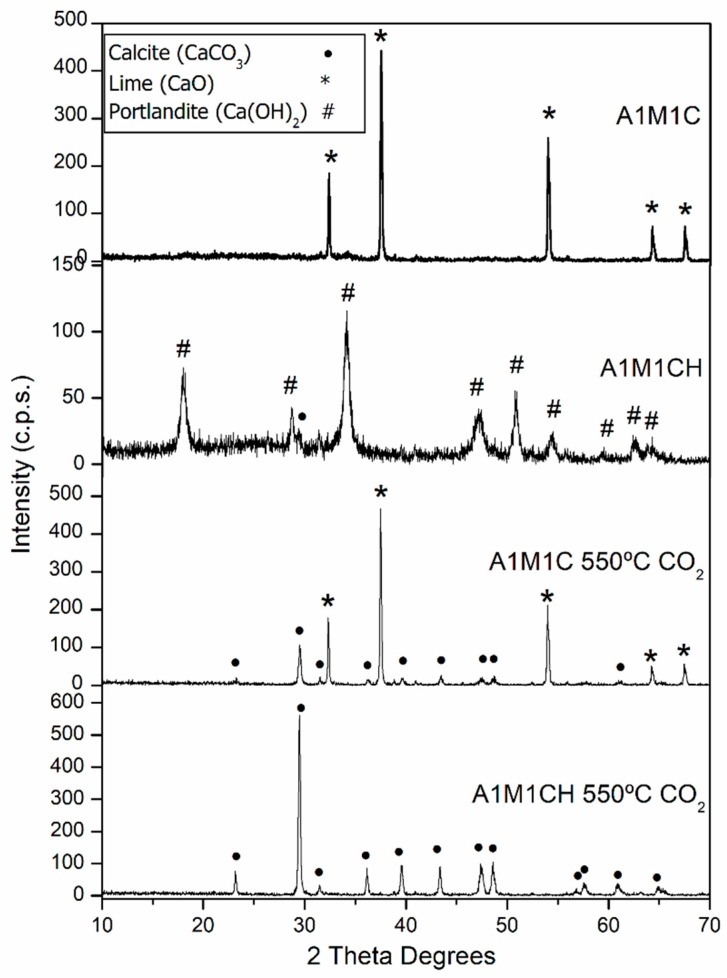
X-ray diffraction (XRD) patterns of the samples A1M1C and A1M1CH and the samples A1M1C and A1M1AC heated at 550 °C in a tubular oven with a CO_2_ flow of 20 mL min^−1^.

The fourth test consisted of a study of CO_2_ adsorption in a tubular oven using the calcined A1M1 sample (A1M1C) and calcinated and rehydrated (A1M1CH). The diffractograms (Figure 8) show the presence of CaO in the first and Ca(OH)_2_ (JCPDS: 04-0733) [28] in the second. Once placed in the tubular oven (10 g), a CO_2_ atmosphere was established for which a flow of CO_2_ was passed through the tubular furnace during the whole experiment (1/2 h), and the temperature was raised to 550 °C (non-isothermal conditions), trying to simulate what would happen in an industrial process if the adsorbent is put in contact with a stream of CO_2_. After 30 min, as reflected in the X-ray diffraction patterns (Figure 8), only in the calcined and rehydrated sample was the carbonation process total, since all the X-ray reflections correspond to the calcite phase (CaCO_3_). The carbonation reaction includes two simultaneous processes, elimination of water to originate CaO and the adsorption of CO_2_ in the newly created CaO surface (Equation (1)).

In the experimental conditions used, the activation energy is not sufficient to cause the total carbonation of CaO, since higher temperatures are needed [35], and instead are only sufficient to carbonate the Ca(OH)_2_ at the same moment it decomposes to CaO (481 °C). This is in agreement with the studies of Materic, et al. [36] who indicated that solid-gas carbonation was much greater when Ca(OH)_2_ was directly carbonated at intermediate temperatures, compared to carbonation of CaO. On the other hand, when the reaction occurs at lower temperatures, the risk of sintering is very low [43], with the subsequent recyclability of the adsorbent for further cycles.

The weight increases for sample A1M1CH, treated in a tubular oven, was 34.11%, very close to the theoretical capacity of 35.11% and the adsorption capacity was 577.00 mg g^−1^ close to the theoretical maximum of 593.95 mg g^−1^. This result is in agreement with those reported by Montes-Hernández, et al. [35], proposing a temperature greater than 300 °C for the carbonation reaction. These authors point out that at temperatures lower than 600 °C under isothermal conditions they never reach total carbonation levels using Ca(OH)_2_ nanoparticles and attribute it to the formation of dense, non-porous sheets of mineral carbonate around the particles of Ca(OH)_2_. Under non-isothermal conditions they achieve total carbonation at temperatures close to 600 °C, as already mentioned in relation to the simultaneous exit of H_2_O and carbonate formation (Blamey et al. [37]).

The experiment was repeated 10 times with the same sample A1M1, calcined and rehydrated. The calcite phase (CaCO_3_) is the only phase present after each carbonation cycle. No appreciable losses of adsorption capacity were observed. This is in agreement with Nikulshina et al. [44] who reported that after five cycles of carbonation-calcination, the loss of adsorption capacity was 0.1%. The carbonation was carried out at 365–400 °C and the calcination at 800–875 °C and virtually no sintering occurs.

In summary, it can be concluded that limestone samples of average particle size <30 µm are the ones that have the most favourable size for CO_2_ capture. The previous hydration of the oxides formed, and the temperature of 550 °C, favours the CO_2_ capture process.

## 4. Conclusions

This work has analyzed the influence of particle size of a calcite from a quarry on the efficiency of CO_2_ capture. The final objective is its application in the elimination of CO_2_ from gases emitted by coal-fired power plants. Three different size fractions of particle <500 µm, <100 µm, and <45 µm have been studied. Calcite, calcined calcite and calcined and rehydrated calcite were used and were characterised by means of X-ray diffraction, N_2_ adsorption isotherms, thermogravimetric analysis, transmission electron microscopy and particle size by laser diffraction. The calcination process had little effect on the particle size of the smaller samples A1045 and A1M1 (<30 μm). The N_2_ and CO_2_ isotherms showed a very important increase in the surface of the calcined and rehydrated samples (A15CH, A1045CH, and A1M1CH) with respect to the calcinated or pristine samples. Likewise, the same value of average pore diameter (3.97 nm) was observed in the samples A045CH and A1M1CH. 

The results of the CO_2_ isotherms show that the capacity of physical adsorption of CO_2_ using calcite, calcined calcite or calcined and rehydrated calcite is very low, although the particle size was small (<30 μm). The best results were obtained for the sample A1M1CH (5.3 mg of CO_2_ g^−1^ of adsorbent). Even when the pressure is increased to 23 bars of CO_2_, the capture is low, the sample A1M1C adsorbed 7.9 mg of CO_2_ g^−1^ of adsorbent. This same sample at the pressure of 1 bar only captured 2.3 mg of CO_2_ g^−1^ of adsorbent. By contrast, chemical capture at high temperature was more efficient. Regarding calcined samples exposed to a current of CO_2_, the largest adsorption of CO_2_ was for the sample with the smallest particle size A1M1C (330.5 mg of CO_2_ g^−1^ of adsorbent). In calcined and partially rehydrated calcites the adsorption improves, the total adsorption of CO_2_ was greater in the sample A1M1CH (368.44 mg of CO_2_ g^−1^ of adsorbent). The repeated calcination at 900 °C, without previous hydration steps, reduces the capacity of CO_2_ adsorption considerably. This effect was less pronounced when the particle size was smaller, where the sample A1M1C, after 30 cycles, retained an adsorption capacity of 30 mg of CO_2_ g^−1^ of adsorbent, higher than that of sample A15C, which was only 4.3 mg of CO_2_ g^−1^ of adsorbent. Finally, when the adsorption of CO_2_ was carried out in a tubular furnace at 550 °C, just at the moment when the Ca(OH)_2_ was decomposed, the adsorption was practically total (577 mg of CO_2_ g^−1^ of adsorbent). When the calcined sample is completely rehydrated after each cycle, the loss of adsorption capacity is practically negligible, thus improving the recyclability of the adsorbent for subsequent cycles. 

## Figures and Tables

**Table 1:** Physicochemical properties of pristine calcites, calcined and rehydrated.

Material	S_BET_^a^ (m^2^ g^−1^)	S_mp_^b^ (m^2^ g^−1^)	V_p_^c^ (10^−2^ cm^3^ g^−1^)	D_p_^d^ (nm)	S_eq_^e^ (m^2^ g^−1^)
A15	2.7	0	0.82	2.89	10.8
A15C	4.4	0	0.99	2.89	12.9
A15CH	21.3	0	8.84	3.79	18.4
A1045	4.7	0	1.72	2.77	10.9
A1045C	4.5	0	1.16	2.77	11.9
A1045CH	13.8	0	5.70	3.97	15.4
A1M1	3.1	0	0.88	2.89	10.4
A1M1C	3.9	0	0.92	3.17	12.1
A1M1CH	18.9	0	5.87	3.97	19.1

^a^ BET specific surface area determined in the range of relative pressures from 0.05 to 0.2; ^b^ Micropore area, calculated by t-Plot method. ^c^ Single-point pore volume; ^d^ Diameter pore average size, calculated from the adsorption branch according to the BJH method; ^e^ Calculated from CO_2_ isotherm.

**Table 2:** Gas-solid carbonatation of different samples under non-isothermal conditions at the same heating rate (5 °C min^−1^).

Sample	Δweight (%)	Δweight ^a^ (%)	Total Loss of Water (%)	Total loss (%)	Ca(OH)_2_ (%)	CaO ^b^ (%)	CaO ^c^ (%)
A15	−41.81	−44.00	-	−41.81	-		-
A15C	+26.66	+78.48	-	−26.66	-	100.00	33.97
A15CH	+26.07	+64.68	−7.72	−33.79	31.80	68.20	27.85
A1045	−41.12	−44.00	-	−41.12	-		-
A1045C	+32.98	+78.48	-	−32.98	-	100.00	33.51
A1045CH	+25.64	+66.88	−6.50	−32.14	26.75	73.25	28.28
A1M1	−41.86	−44.00	-	−41.86	-		-
A1M1C	+33.05	+78.48	-	−33.05	-	100.00	42.11
A1M1CH	+28.39	+63.39	−8.45	−36.84	34.80	65.20	31.61

^a^ Theoretical value; ^b^ CaO total; ^c^ CaO reacted from total of CaO.
